# Delayed Captopril Administration Mitigates Hematopoietic Injury in a Murine Model of Total Body Irradiation

**DOI:** 10.1038/s41598-019-38651-2

**Published:** 2019-02-18

**Authors:** Elizabeth A. McCart, Young H. Lee, Jyoti Jha, Ognoon Mungunsukh, W. Bradley Rittase, Thomas A. Summers, Jeannie Muir, Regina M. Day

**Affiliations:** 1 Department of Pharmacology and Molecular Therapeutics, Uniformed Services University of the Health Sciences, Bethesda, MD 20814 USA; 2 Altimmune, Inc., Gaithersburg, MD 20878 USA; 3 Rise Therapeutics, Rockville, MD 20850 USA; 4 Department of Anesthesia, Uniformed Services University of the Health Sciences, Bethesda, MD 20814 USA; 5 Department of Pathology, Uniformed Services University of the Health Sciences, Bethesda, MD USA

## Abstract

The increasing potential for accidental radiation exposure from either nuclear accidents or terrorist activities has escalated the need for radiation countermeasure development. We previously showed that a 30-day course of high-dose captopril, an ACE inhibitor, initiated 1–4 h after total body irradiation (TBI), improved Hematopoietic Acute Radiation Syndrome (H-ARS) and increased survival in mice. However, because of the time likely required for the deployment of a stockpiled radiation countermeasure to a radiation mass casualty site, there is a need for therapies that can be administered 24–48 hours after initial exposure. Using C57BL/6 mice exposed to an LD_50-80/30_ of ^60^Co TBI (7.75–7.9 Gy, 0.615 Gy/min), we show that low-dose captopril administration, initiated as late as 48 h post-TBI and continued for 14 days, significantly enhanced overall survival similarly to high-dose, rapid administration. Captopril treatment did not affect radiation-induced cell cycle arrest genes or the immediate loss of hematopoietic precursors. Reduced mortality was associated with the recovery of bone marrow cellularity and mature blood cell recovery at 21–30 days post-irradiation. Captopril reduced radiation-induced cytokines EPO, G-CSF, and SAA in the plasma. Finally, delayed captopril administration mitigated brain micro-hemorrhage at 21 days post-irradiation. These data indicate that low dose captopril administered as late as 48 h post-TBI for only two weeks improves survival that is associated with hematopoietic recovery and reduced inflammatory response. These data suggest that captopril may be an ideal countermeasure to mitigate H-ARS following accidental radiation exposure.

## Introduction

There is currently an elevated potential for accidental radiation exposure due to increased usage of nuclear power, increased medical and industrial applications, and the heightened potential for nuclear terrorism or war^1–3^. Only two drugs are currently approved by the US Food and Drug Administration (FDA) for prophylactic radioprotection in clinical settings, amifostine and palifermin, and only three agents, two G-CSF preparations (filgrastim and pegfilgrastim) and one GM-CSF preparation (sargramostim), have been approved for protection against Hematopoietic Syndrome of Acute Radiation Syndrome (H-ARS)^4^. Thus there is an urgent need to develop countermeasures to enhance survival after radiation exposure, and especially to discover agents with excellent safety profiles that can be easily distributed and administered.

The hematopoietic system is uniquely sensitive to radiation damage, including the mature blood cells and hematopoietic stem cells in the bone marrow compartment that are critical for blood cell regeneration^5,6^. Total body radiation exposure results in mortality within 30 days, typically from hematopoietic insufficiency, including severe anemia and leukopenia that impairs immune function, allowing life-threatening opportunistic infection, and increased vascular permeability and hemorrhage in vital organs^3,7,8^. The sensitivity of the immune system to radiation is not completely understood, but is believed to be due to the rapid proliferative rates and reduced DNA repair capacity of myeloid/lymphoid hematopoietic progenitors^8–10^. Compared to quiescent cells or cells with low proliferative rates, rapidly cycling cells display increased DNA damage from radiation, resulting in higher levels of apoptosis and/or senescence^6,11,12^. Agents that transiently arrest the hematopoietic stem cells (HSC) in the G_0_/G_1_ phases of the cell cycle can reduce radiation-induced genotoxicity, senescence, and stem cell pool exhaustion^13,14^.

Elements of the renin-angiotensin system (RAS), especially angiotensin II (Ang II) and angiotensin converting enzyme (ACE), were demonstrated to be effective targets for mitigation of H-ARS^15–17^. The RAS plays a key role in the regulation of blood pressure and blood volume homeostasis^18^, but components of this system also regulate the proliferation and maturation of hematopoietic cells^19^. Ang II directly modulates the development and proliferation of hematopoietic progenitor cells (HPC) through Ang II receptors expressed on the cell surface^19–23^. Ang II also indirectly regulates hematopoiesis through the regulation of other hematopoietic cytokines such as erythropoietin^17,24–26^. Additionally, ACE, the metalloprotease required for the proteolytic activation of Ang II, regulates other peptides with hematopoietic activities such as substance P, Ac-SDKP, and angiotensin 1-7^27^. Thus, drugs that affect the RAS can have widespread effects, both directly and indirectly, on hematopoietic cell development and proliferation.

We and others have shown that ACE inhibition can reduce the severity of H-ARS in murine models^15–17^. We previously demonstrated that captopril (110 mg/kg/day) allowed 100% survival from an LD_50/30_ dose of radiation in mice when administered from 1–4 h following radiation exposure through 30 days post-irradiation^16^. We demonstrated that at the LD_50/30_ dose of radiation, captopril improved bone marrow and blood cell recovery, including red blood cells, reticulocytes, and platelets. Using sublethal levels of radiation to allow sufficient recovery of hematopoietic progenitors, we found that captopril treatment improved recovery of colony forming units-granulocyte macrophage (CFU-GM) and –megakaryocyte (CFU-M), and total colony forming units. Captopril administration was associated with transient growth arrest of hematopoietic progenitors and suppression of radiation-induced EPO expression^16,17^. In this study, we investigated a reduced dosage of captopril and a delayed, shortened time course of administration following higher levels of acute radiation exposure. We found that 13 mg/kg/day captopril administered from 48 h through 2 weeks post-irradiation mitigated radiation H-ARS, as characterized by bone marrow repopulation and mature blood cell recovery. Captopril also mitigated radiation-induced surges in cytokine expression and reduced vascular dysfunction following ionizing radiation exposure.

## Results

### Delayed captopril administration improves 30-day survival following 7.9 Gy total body irradiation

**Figure 1:**
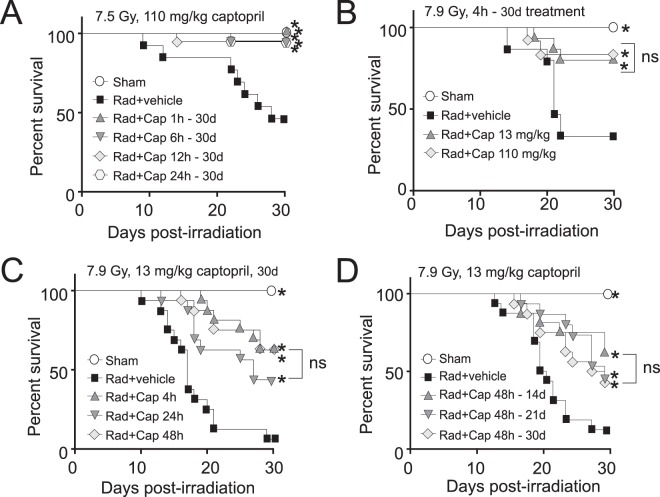
Kaplan-Meier Curves of the effects of reduced dosage and delayed administration of captopril on survival from total body irradiation. C57BL/6 mice, 12–14 weeks of age, were exposed to total body ^60^Co irradiation (0.6 Gy/min) or sham irradiated (sham). Irradiated (Rad) mice either received vehicle (Veh) or captopril (Cap), provided in the drinking water. For all irradiated groups, n = 16–20; for the sham irradiated group, n = 8. The percentage of surviving mice are shown. (**A**) Mice were exposed to 7.5 Gy (LD_50/30_) total body irradiation and were treated with either vehicle only (water) or with captopril administration (110 mg/kg/day) beginning at various time points from 1 h to 24 h postirradiation, as indicated, through 30 days postirradiation. (**B**) Mice were exposed to 7.9 Gy. Mice were untreated or captopril (110 mg/kg/day or 13 mg/kg/day) was administered 4 h through 30 days post-irradiation. (**C**) Mice were exposed to 7.9 Gy. Mice were untreated or captopril (13 mg/kg/day) was initiated at either 4 h, 24 h, or 48 h post-irradiation through 30 days post-irradiation. (**D**) Mice were exposed to 7.9 Gy. Mice were untreated or captopril (13 mg/kg/day), administered in the drinking water, was initiated at 48 h post-irradiation through 14, 21 or 30 days post-irradiation. *p < 0.05 compared with radiation + vehicle.

Our previous work demonstrated that 110 mg/kg/day captopril, administered from 1 h through 30 days post-irradiation, resulted in 100% survival in C57BL/6 J mice exposed to an LD_50/30_ dose of ^60^Co total body irradiation (TBI)^16^. However, rapid administration of radiation countermeasures may not be feasible following a large-scale radiation event, due to the time required to both transport medication to a mass casualty site and administer it to a large population. We therefore investigated the effect of delayed administration of 110 mg/kg/day captopril on the survival of mice exposed to 7.5 Gy TBI and found that delaying the initial dose of captopril up to 24 h post-TBI was protective from radiation-induced death (Fig. 1A). At 7.5 Gy, mice that did not receive captopril had a 47.5% survival. In contrast, mice treated with captopril 1, 6, 12 or 24 h post-irradiation showed a significantly increased survival rate of 100%, 90%, 95%, and 90%, respectively (Log-Rank tests, p < 0.05 compared with radiation + vehicle). There was no statistical difference in survival rates among the captopril treated groups. These data indicated that captopril administration as late as 24 h did not significantly reduce survival compared to treatment 1 h post-TBI.

We then investigated the effect of reducing the captopril dose on survival, using 13 mg/kg/day, to determine whether the lower dosage of captopril would have protective effects. Additionally, the radiation exposure was increased to 7.9 Gy to induce greater mortality to better distinguish any subtle differences between the captopril treatment groups. Vehicle-treated mice irradiated with 7.9 Gy had 31% survival at 30 days (Fig. 1B). Mice treated with captopril (110 or 13 mg/kg/day) starting 4 h post-TBI exhibited significant survival improvement, displaying 83% and 75% survival for high- and low-dose captopril, respectively (Log-Rank test, p < 0.05 for both groups vs radiation + vehicle). Furthermore, there was no significant difference in survival between the groups of mice treated with high-dose versus low-dose captopril. These data demonstrate that lower dosages of captopril are sufficient to provide protection against acute hematopoietic injury and improve overall survival.

We next tested whether the initial administration of low-dose captopril (13 mg/kg/day) could be delayed following TBI, as we had observed for high-dose captopril (Fig. 1C). Following 7.9 Gy TBI, mice were treated with vehicle or 13 mg/kg/day captopril for 30 days, with the treatment initiated at various times post-irradiation (Fig. 1C). Irradiated, vehicle-treated mice displayed 6.25% survival at 30 days. Mice treated with captopril, initiated at 4, 24, or 48 h post-irradiation, displayed significant increases in survival, with 62%, 42%, and 62% survival, respectively (Log-Rank test, p < 0.0001 radiation + vehicle vs radiation + captopril 4 h; p < 0.05 radiation + vehicle vs radiation + captopril 24 h; and p < 0.0001 radiation + vehicle vs radiation + captopril 48 h). There was no statistical difference among the captopril treated groups. These data show that the initial administration of low-dose captopril could be delayed as long as 48 hours post-TBI without changing its efficacy.

We investigated whether the length of low-dose captopril administration could be reduced. Irradiated mice were treated with vehicle or with captopril initiated at 48 h post-TBI through 14, 21 or 30 days post-irradiation (Fig. 1D). Irradiated vehicle-treated mice displayed only 12% survival. Mice treated with captopril through 14, 21 or 30 days post-irradiation all had a significant increase in survival, with 62%, 43% and 43% survival, respectively (Log-Rank tests, p < 0.05 for all groups vs radiation + vehicle). There was no significant difference in survival among captopril treatment groups. Together these data suggest that low-dose captopril administration from days 2–14 post-irradiation provided protection from radiation induced hematopoietic injury equivalent to high-dose captopril treatment initiated 1–4 h post-irradiation through 30 days post-irradiation.

### Delayed captopril administration improves peripheral blood cell recovery following 7.9 Gy irradiation

**Figure 2:**
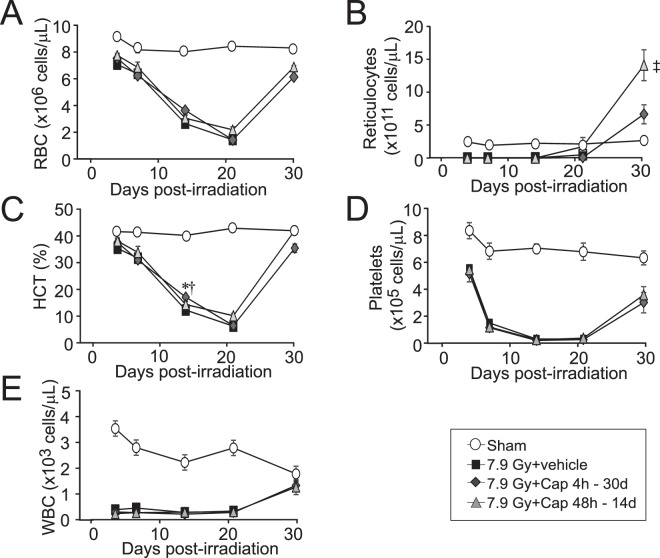
Effect of delayed captopril treatment on mature blood cell loss and recovery after total body irradiation. C57BL/6 mice, 12–14 weeks of age, were exposed to 7.9 Gy total body ^60^Co irradiation (0.6 Gy/min). Mice received vehicle or received captopril (13 mg/kg/day), administered in the drinking water either 48 h through 14 days post-irradiation or 4 h through 30 days post-irradiation. Blood was obtained at 3, 7, 14, 21 and 30 days post-irradiation for analysis and quantification of (**A**) red blood cells (RBC), (**B**) reticulocytes, (**C**) hematocrit (HCT), (**D**) platelets, and (**E**) peripheral white blood cells (WBC). Data show means ± standard error of the mean, n = 4–5 mice per group. *p < 0.05 for captopril treatment 4 h–30 d vs radiation + vehicle; ^†^p < 0.05 for captopril treatment 48 h–14 d vs radiation + vehicle. ^‡^indicates a significant increase for captopril 48 h–14 d vs sham, p < 0.05.

We previously reported that high-dose captopril treatment, initiated 1 h following TBI, mitigated radiation ablation of some peripheral blood cell populations^16^. We assessed the effects of low-dose captopril, administered either 4 h – 30 days or 48 h – 14 days post-irradiation, on the loss of peripheral blood cell populations. 7.9 Gy TBI resulted in significant decrease of all mature blood cell types as early as 4 day post-irradiation compared to the sham-irradiated group, including red blood cells (RBC), reticulocytes, platelets and white blood cells (WBC) (Fig. 2A–E). Nadirs in RBC (1.4 × 10^6^ ± 0.1 × 10^6^ cells/µl), hematocrit (HCT) (6.7 ± 0.7%), and platelets (29.5 × 10^5^ ± 6.6 × 10^5^ cells/µl) occurred ~21 days post-irradiation in all animals. Reticulocytes and WBC numbers declined by post-irradiation day 4 (1.6 × 10^11^ ± 0.3 × 10^11^ cells/µl, and 0.3 × 10^3^ ± 0.04 × 10^3^ cells/µl, respectively), with reticulocytes showing some improvement in untreated mice at day 21 post-irradiation (52 × 10^11^ ± 37 × 10^11^ cells/µl). Because no animals survived past day 25 in the irradiated, vehicle-treated cohort, no values were obtained after that time point. Treatment with captopril did not significantly affect radiation-induced mature blood cell reduction for any blood cell type, and nadirs were similar for all irradiated groups. We did observe a modest improvement in the hematocrit in both captopril treated groups at 14 days post-irradiation (Fig. 2C, p ≤ 0.05), but the nadirs of the hematocrits were not significantly different from that of the vehicle-treated irradiated mice. However, by 30 days post-irradiation, both captopril treated groups had similar levels of RBC (6.2 vs 6.9 × 10^6^ ± 0.2–0.3 × 10^6^ cells/µl), HCT (36 vs 43 ± 1.5–1.6%), platelet (304 vs 363 × 10^5^ ± 76–62 × 10^5^ cells/µl), and total WBC (1.3 vs 1.2 × 10^3^ ± 0.2–0.3 × 10^3^ cells/µl). Furthermore, mice that received delayed captopril administration (48 h) showed enhanced reticulocyte recovery compared to mice treated 4 h following irradiation, and both groups showed recovery of reticulocytes above basal levels (668 vs 1414 × 10^11^ ± 145–236 × 10^11^ cells/µl, for 4 h – 4 weeks and 48 h – 2 weeks respectively, p ≤ 0.05) suggesting that all captopril-treated mice were on a course of robust blood cell recovery. No direct comparison of blood cell recovery can be made between captopril- and vehicle-treated mice, as no vehicle-treated mice survived to the 30 day time point. Recovery of blood cells in the captopril-treated groups correlated with improved survival.

### Delayed captopril administration improves bone marrow cellularity and allows recovery from cell cycle checkpoint gene expression following 7.9 Gy total body irradiation

**Figure 3:**
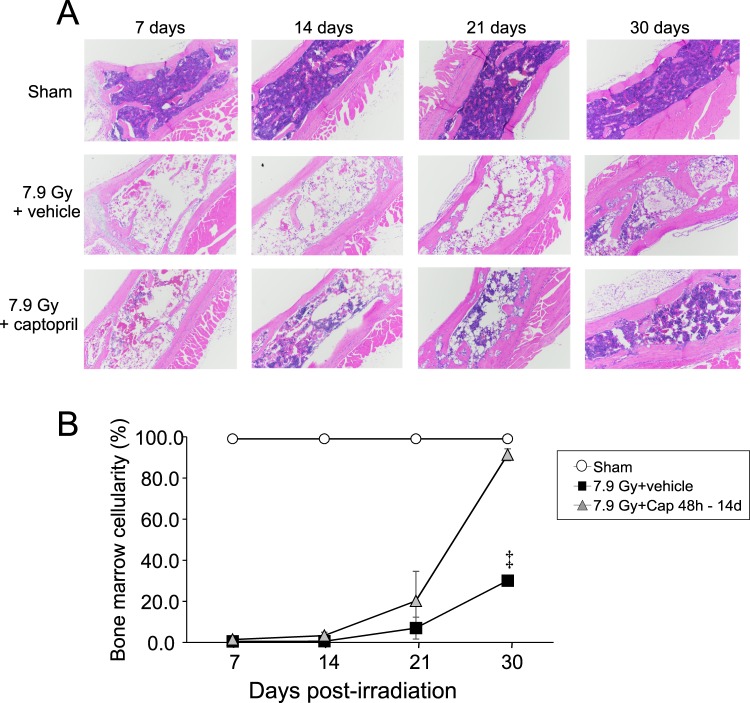
Effect of delayed captopril treatment on bone marrow cellularity following total body irradiation. C57BL/6 mice, 12–14 weeks of age, were exposed to 7.9 Gy total body ^60^Co irradiation (0.6 Gy/min) or sham irradiated (sham). Mice received vehicle (7.9 Gy + vehicle) or received captopril (13 mg/kg/day, 7.9 Gy + Cap), administered in the drinking water either 48 h through 14 days post-irradiation. (**A**) Sternabrae were obtained from mice at the indicated time points and processed for H&E staining. (**B**) Bone marrow cellularity was scored by a hematological pathologist blinded to the treatment groups, n = 3–5 per group, except for the 30 day time point for radiation + vehicle, which had only one animal (indicated by^†^).

Our laboratory showed that captopril, administered 1 h - 30 days post-irradiation, resulted in improved bone marrow recovery, especially colony forming units-granulocyte macrophage, macrophage, and total colony forming units^16^. We further demonstrated that the recovery of these hematopoietic populations was preceded by a pause in the cell cycle induced by captopril^16^. We investigated the effect low-dose, delayed captopril administration on bone marrow recovery. Sternabrae cellularity was assessed and scored blind by a pathologist in sham irradiated mice, or in mice receiving 7.9 Gy TBI and either vehicle-treated or low-dose captopril-treated (Fig. 3, Table S1). Sham irradiated, vehicle-treated mice showed 99% cellularity over the entire 30 day experimental period. Vehicle-treated mice exposed to 7.9 Gy TBI displayed a significant decrease in cellularity in the sternabrae compared to the sham irradiated mice, showing <1% cellularity on days 7 and 14, which rose to ~7% on day 21, and then to ~30% at day 30 in the one surviving animal in this group. Captopril-treated mice displayed the same nadir of bone marrow cellularity at 7–14 days post-irradiation as vehicle-treated animals. However, the bone marrow of mice treated with captopril from 48 h - 14 days post-irradiation showed ~20% cellularity at 21 days post-irradiation and ~90% cellularity by 30 days post-TBI. These data demonstrate that low-dose captopril, administered 48 h through 14 days post-TBI displayed the capacity for almost full bone marrow recovery by 30 days post-irradiation.

**Figure 4:**
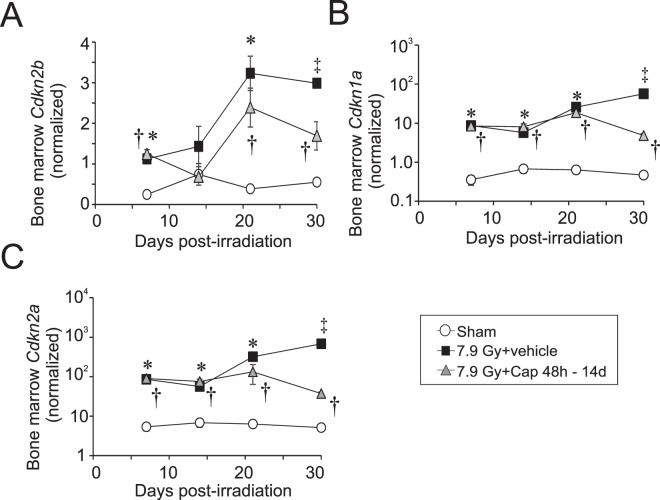
Effect of delayed captopril treatment on senescent-associated gene expression in the bone marrow following total body irradiation. C57BL/6 mice, 12–14 weeks of age, were exposed to 7.9 Gy total body ^60^Co irradiation (0.6 Gy/min) or sham irradiated (sham). Mice received vehicle (7.9 Gy + vehicle) or received captopril (13 mg/kg/day, 7.9 Gy + Cap), administered in the drinking water 48 h – 14 days post-irradiation. Bone marrow was obtained at the indicated times and RT-qPCR was performed to quantify mRNA for the following cell cycle genes: (**A**) *Cdkn2b*; (**B**) *Cdkn1a*; (**C**) *Cdkn2a*. Data show means ± SEM, n = 3–5 per group, except for the 30 day time point for radiation + vehicle, which had only one animal (indicated by ^‡^). *Indicates p < 0.05 between radiation + vehicle and sham; ^†^indicates p < 0.05 between radiation + captopril and sham.

Our previous studies demonstrated that TBI induced the expression of cyclin-dependent kinase inhibitors in the bone marrow in response to DNA damage, potentially associated with cell cycle arrest in senescence^13^. To determine whether delayed captopril treatment suppressed cell cycle checkpoint gene expression, bone marrow was obtained from sham controls or irradiated mice treated with either vehicle or low-dose captopril from 48 h–14 days post-irradiation. RT-qPCR was performed to determine levels of *Cdkn2b* (p15), *Cdkn1a* (p16), and *Cdkn2a* (p21) mRNA. Radiation treatment resulted in a significant increase of *Cdkn2b*, *Cdkn1a*, and *Cdkn2a* expression over the time course of the experiment, beginning 7 days post-irradiation (Fig. 4). Captopril treatment did not significantly suppress radiation-induced expression of any of these genes over days 7–21. A decrease in all three genes was observed on day 30 in radiation + captopril mice compared with radiation + vehicle, but significance could not be determined as only one irradiated, vehicle-treated mouse survived until day 30 (Fig. 4). The observed decrease in the *Cdkn* genes in the captopril treated animals from day 21–30 post-irradiation correlates with the recovery of bone marrow cellularity observed at over this time with captopril (Fig. 3).

### Captopril modulates radiation-mediated increase of EPO and pro-inflammatory cytokines G-CSF and SAA

**Figure 5:**
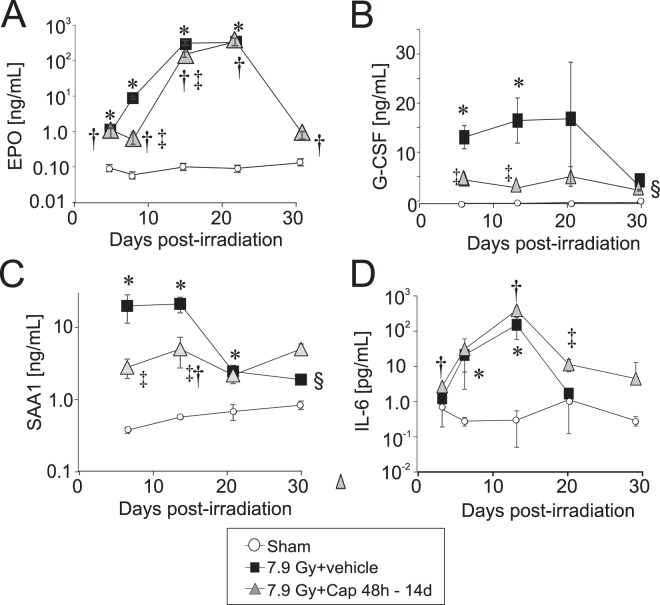
Effect of delayed captopril administration on cytokine levels in peripheral blood following total body irradiation. C57BL/6 mice, 12–14 weeks of age, were exposed to 7.9 Gy total body ^60^Co irradiation (0.6 Gy/min) or sham irradiated (sham). Mice received vehicle (7.9 Gy + vehicle) or received captopril (13 mg/kg/day, 7.9 Gy + Cap), administered in the drinking water 48 h – 14 days post-irradiation. Serum was obtained at the indicated times, and the following growth factors and cytokines were quantified by MSD or ELISA: (**A**) EPO; (**B**) G-CSF; **C**. SAA1; (**D**) IL-6. Data show means ± SEM, n = 3–5 per group, except for the 30 day time point for radiation + vehicle, which had only one animal (indicated by ^§^). *Indicates p < 0.05 between radiation + vehicle and sham; ^†^indicates p < 0.05 between radiation + captopril and sham; ^‡^indicates p < 0.05 between radiation + vehicle and radiation + captopril.

We previously demonstrated that radiation-induced loss of RBC and reduced hematocrit was associated with the induction of hypoxia-inducible factors (HIF) in the kidney and increased expression of EPO^14,16^. We also demonstrated that high-dose captopril, administered within 1–4 h post-irradiation, mitigated radiation-induced loss of RBC, and suppressed HIF activation and EPO expression^14,16,17^. We hypothesized that the suppression of radiation-induced cytokines such as EPO protects hematopoietic progenitors from the induction of rapid cycling that can lead to stem cell pool exhaustion^16^. We therefore investigated the effect of 48 h delayed captopril administration on EPO expression following 7.9 Gy TBI. At 4 days post-irradiation, irradiated mice with or without captopril displayed a ~10-fold increase in serum EPO (Fig. 5A). At 7 and 14 days post-irradiation, captopril reduced the radiation-induced surge in EPO levels (7 days: radiation + vehicle = 8.66 ± 0.9 ng/ml; radiation + captopril = 0.57 ± 0.7 ng/ml; 14 days radiation + vehicle = 288 ± 140 ng/ml; radiation + captopril = 140 ± 24 ng/ml). However, both irradiated groups had similarly elevated EPO at day 21 post-irradiation. Serum EPO declined sharply in captopril-treated animals by day 30 post-irradiation; no vehicle-treated irradiated animals were alive for comparison at this time point.

Several studies have demonstrated that other hematopoietic cytokines and growth factors are dramatically increased in response to TBI, including G-CSF, SAA, IL-6, SCF, MIP1a and 1b, MCP1, FLT-3, IL-10, IL-1β, IL-2, KC/CXCL1^28^. The observed increases in specific cytokines and growth factors are believed to play a role in natural resistance to radiation damage and may be involved in hematopoietic recovery as well as inflammatory responses after radiation^28–30^. We investigated the effect of 48 h delayed administration of low-dose captopril on these cytokines and growth factors following 7.9 Gy TBI.

We observed significant TBI-induced increase in G-CSF and SAA that was modulated by delayed captopril treatment. Irradiation caused a ~40-fold increase in G-CSF at 7–21 days post-irradiation (Fig. 5B). Captopril treatment significantly suppressed radiation induced G-CSF at days 7 and 14 post-irradiation (p < 0.05). Similarly, irradiation caused a ~50-fold increase in SAA1 within 7 days of 7.9 Gy TBI (Fig. 5C). Delayed, low-dose captopril treatment significantly attenuated radiation-induced SAA1 levels on days 7 and 14 post-irradiation (p < 0.05). Note that at 30 days post-irradiation, only one animal remained in the irradiated vehicle-treated group, and statistical significance could not be determined.

IL-1β and IL-6 are primary regulators of SAA1 following acute injury^31,32^. We did not detect significant elevation of IL-1β over the time course examined (data not shown), so we examined the effect of captopril on IL-6 post-irradiation. Radiation significantly increased IL-6 levels on days 7 and 14 post-irradiation, but captopril treatment did not significantly suppress IL-6 at any time points (Fig. 5D). These data suggest that captopril does not suppress radiation-induced SAA1 through the regulation of either IL-1β or IL-6.

We also observed that radiation significantly increased serum levels of SCF, MIP1α, MCP1 and FLT-3, but were not significantly affected by captopril (Fig. S1). We also did not observe significant changes in MIP-1b, IL-2, IL-10, or KC levels in any treatment groups at the time points that we examined for this study (data not shown). Together these data suggest that captopril has specific effects on systemic inflammation following total body irradiation.

### Delayed captopril administration mitigates radiation-induced brain micro-hemorrhage

**Figure 6:**
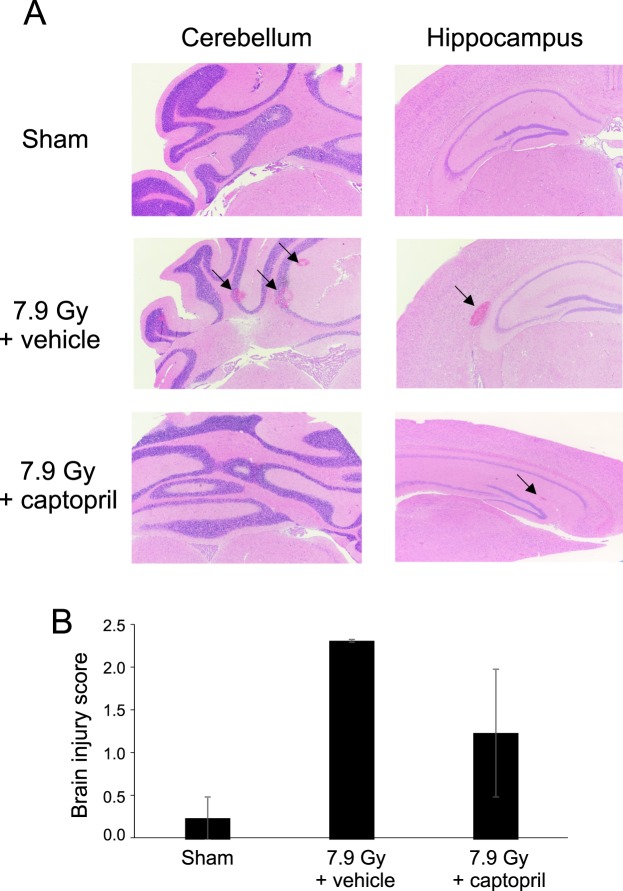
Captopril reduces brain hemorrhage following 7.9 Gy total body irradiation at 21 days post-irradiation. C57BL/6 mice, 12–14 weeks of age, were exposed to 7.9 Gy total body ^60^Co irradiation (0.6 Gy/min) or sham irradiated (sham). Mice received vehicle (7.9 Gy + vehicle) or received captopril (13 mg/kg/day, 7.9 Gy + Cap), administered in the drinking water 48 h – 14 days post-irradiation. At 21 days days post-irradiation, brains were obtained after euthanasia and fixed for histology. (**A**) Representative histological sections of the cerebellum and hippocampus from mice. (**B**) Brain injury scores, performed by a pathologist blinded to the treatment group. Bar graphs show means ± SEM, n = 3–4 per group

Survival following TBI is linked primarily to mature blood cell and bone marrow recovery, but hemorrhage due to clotting insufficiency, potentially related to loss of platelets, and endothelial dysfunction can be a major factor for morbidity and mortality in larger mammals and humans following radiation exposure^7^. We previously showed that high-dose administration of captopril from 4 h through 30 days post-irradiation reduced brain micro-hemorrhages in mice exposed to 7.5 Gy TBI^16^. We therefore assessed whether delayed, low-dose captopril could similarly mitigate gross vascular or microvascular hemorrhage at 21 days post-irradiation in brains of mice exposed to 7.9 Gy TBI. No overt brain hemorrhage was visible in sham-irradiated mice, but radiation induced significant brain micro-hemorrhaging (Fig. 6A). Gross hemorrhages were also observed on the brain surfaces (Fig. S2). Histologic sections from blinded samples were scored by a pathologist using criteria for level 1–4 hemorrhage severity to assess whether captopril treatment reduced radiation-induced hemorrhage (Fig. 6B). Only one of four sham-irradiated brains showed any evidence of hemorrhage (μ = 0.25; scores of 0, 0, 0, and 1 for four individuals). Vehicle-treated, irradiated animals showed significant signs of hemorrhage (μ = 2.3; scores of 1, 3, and 3 for three individuals). Captopril reduced the average hemorrhage severity (μ = 1.25; scores of 0, 2, 0, and 3 for four individuals), with two out of four captopril treated mice displaying no hemorrhage and two displaying significant hemorrhages. These data suggest a trend where delayed, low-dose captopril treatment reduces pathologic brain hemorrhages.

## Discussion

Due to the increasing potential of radiation exposure to large populations, either from nuclear accidents or terrorist activities, there is a need for the development of safe, easily administered, and readily available radiation countermeasures. The optimal timing of both the initial dosing and the overall duration of treatment for radiation countermeasures has been a topic of debate. In the case of a mass casualty event, emergency response teams may encounter delays in the administration of radiation countermeasures, and current guidelines from the National Institute of Allergy and Infectious Diseases support the development of radiation countermeasures with efficacy when administered 24 h or later following radiation exposure^33,34^. Our current study interrogates the effective dosages of captopril as well as the time course of administration required for mitigation of radiation injury. We previously showed that a 30 day course of the ACE inhibitor captopril (110 mg/kg/day), first administered 1–4 h post-irradiation, mitigated H-ARS in mice^16,17^. In our current study we show that 13 mg/kg/day mitigates H-ARS in mice to the same degree as the higher dose. Strikingly, our current data also demonstrate that captopril administration can be delayed for up to 48 h post-irradiation and that the time course of administration can be reduced to as little as two weeks. This reduced dosage and delayed administration time mitigated H-ARS in mice by TBI to a level similar to that obtained in our earlier studies. In this study we report robust recovery of bone marrow cellularity and mature blood cell populations in captopril-treated animals within 30 days post-irradiation. It has been demonstrated that mortality from H-ARS within 30 days post-irradiation is mainly due to the effects of hematopoietic insufficiency^2,5,7,35^. Although the irradiated non-treated mice died prior to 30 days post-irradiation making comparisons at that time point impossible, hematopoietic recovery and improved bone marrow cellularity in the treated animals correlated with increased survival. Without hematopoietic recovery, survival would not occur. In addition to blood cell recovery and bone marrow repopulation, we saw the suppression of radiation-induced cytokine expression, and reduced radiation-induced brain hemorrhages. The ability to delay the initial dose to 48 hours post-irradiation, coupled with the wide availability, safety, stability, and ease of dosing with captopril make it an ideal countermeasure to mitigate H-ARS following radiation exposure. Moreover, the efficacy of reduced dosage and shortened time course of administration further minimize side effects of the drug.

Studies of countermeasures for H-ARS have revealed a paradox for the mechanism of action of successful agents: improved survival has been reported for agents that induce hematopoietic progenitor proliferation as well as for agents that induce transient growth arrest of hematopoietic progenitors^13,36,37^. Agents that induce hematopoietic progenitor proliferation, such as G-CSF and GM-CSF (both FDA approved for treatment of H-ARS^4^) or thrombopoietin, increase recovery rates of mature blood cell populations, and are believed to avert mortality by reducing the time at which blood cell populations are at the nadir. This ameliorates hematopoietic insufficiency and combats opportunistic infection, which are thought to be the primary causes of death following total body irradiation. However, a recent report showed that while G-CSF administration reduced H-ARS, it exacerbated long-term bone marrow injury, potentially by exhausting the proliferative capacity of the hematopoietic stem cell pool^38^.

In contrast to pro-proliferative agents, we have shown that countermeasures that induce transient growth arrest in hematopoietic blood cell populations can reduce radiation-induced DNA damage in the bone marrow compartment, possibly by increasing the time for DNA repair^13,39^. When the transient quiescence was relieved, robust recovery was observed for the hematopoietic progenitor and mature blood cell populations^11,13^. We hypothesize that transient quiescence of the bone marrow, accompanied by increased cellular repair, allowed for the subsequent bone marrow and blood cell recovery^13,16^.

The same countermeasure paradox has emerged for modulators of the RAS as radiation countermeasures, since both Ang II peptides and inhibitors of the RAS pathway protect the hematopoietic system from H-ARS^15,40,41^. Ang II acts directly on hematopoietic progenitors^20^ and indirectly through the induction of EPO^17,25,26^ to induce proliferation and survival ameliorating radiation-induced hematopoietic insufficiency. In contrast, we have previously reported that the ACE inhibitor captopril induced transient growth arrest in the hematopoietic compartment, ultimately resulting in improved survival and proliferation of hematopoietic progenitors^14,16,17^. Our current data show that low-dose, delayed captopril administration did not immediately enhance hematopoietic proliferation and repopulation, but was associated with robust delayed recovery of both bone marrow and peripheral blood cellularity by 30-days post-irradiation. The repopulation of the bone marrow compartment correlated with the downregulation of radiation-induced cell cycle arrest genes. Furthermore, the recovery of peripheral blood cell populations and bone marrow cellularity correlated with the reduction of brain micro-hemorrhage, which we previously hypothesized was associated with the prevention of severe thrombocytopenia^16^. Together these data suggest that delayed captopril administration protected sufficient levels of hematopoietic progenitor populations to allow bone marrow recovery leading to full peripheral blood cell recovery.

The actions of captopril may be direct, through the reduction of Ang II signaling on hematopoietic progenitors, as well as indirect, through the modulation of radiation-induced cytokine production. We previously reported that high-dose captopril induced a transient quiescence in the lin^−^ bone marrow progenitor cell populations; however the mechanism(s) responsible for transient quiescence of lin^−^ cells were not identified^16^. In the current study, low-dose, delayed captopril administration mitigated radiation-induced expression of EPO, SAA1, and G-CSF. EPO and G-CSF can stimulate proliferation, survival, and the mobilization of a variety of circulating hematopoietic progenitors^42^. The observed increases in EPO and G-CSF following radiation exposure may be the result of “demand-driven” or “emergency” gene regulation, due to the acute loss of mature granulocytes and RBC^42^. Our findings suggest that the captopril-induced reduction in circulating EPO and G-CSF still allows for repopulation of blood cells post-irradiation.

In contrast to EPO and G-CSF which are hematopoietic cytokines, SAA1 is an acute phase protein, primarily produced by the liver, and elevated in the plasma following trauma, infection, inflammatory reactions, and cancer^31,43^. SAA1 signals through a variety of receptors to regulate downstream pro-inflammatory gene expression^44^. While our current study did not assess the mechanism of SAA1 suppression by captopril, we hypothesize that the suppression may be due to either protection of the liver tissue from radiation damage or suppression of another upstream inflammatory cytokine. Interestingly, SAA1 can induce G-CSF expression^45^, so reduced SAA in captopril treated mice may lead to reduced G-CSF. Further studies are required to improve understanding of the mechanism of radiation-induced expression of inflammatory cytokines.

The development of radiation countermeasures must be performed in accordance with the Animal Rule of the Food and Drug Administration (FDA), which provides guidance for drug development when human efficacy studies are not ethical and field trials are not feasible. The effective dosage of captopril for the prevention of H-ARS in humans is not known. Based on standard calculation methods, 110 mg/kg/day captopril is the human equivalent dose (HED) of ~330 mg/day^46^. While this is below the maximal human dose of 500 mg/day, the average daily human dose of captopril is 37.5–75 mg/day^47^. Based on standard methods for HED calculations, 13 mg/kg/day would be the HED ~39 mg/day, approaching the lowest average daily human dosage. An effective HED for captopril would be calculated following testing in non-human primates or another species accepted by the FDA^48^.

The increasing possibility of accidental radiation exposure has led to an urgent need for the development of radiation countermeasures with excellent safety profiles and with ease of distribution and administration. Our current data show that the ACE inhibitor captopril can be administered up to 48 h post-irradiation at doses equivalent to a normal, safe human dosage for a short time course to prevent of H-ARS in a murine model. ACE inhibitors are used safely and extensively in the general population, and these data provide evidence that the ACE inhibitor captopril may be an ideal candidate radiation countermeasure. Since clinical trials cannot be performed to test the efficacy of radiation countermeasures in human patients^49^, studies to test captopril efficacy in ameliorating H-ARS in in other animal models are essential to determine efficacy this promising radiation countermeasure for the prevention of H-ARS in humans.

## Design and Methods

### Mice

This study was performed in compliance with the recommendations in the Guide for the Care and Use of Laboratory Animals of the National Institutes of Health. The protocol was approved by the Institutional Animal Care and Use Committee of the Armed Forces Radiobiology Research Institute (AFRRI, Bethesda, MD, USA)(Protocol Number: 2015–06–006). All efforts were made to minimize suffering. Female C57BL/6 J mice (The Jackson Laboratory, Bar Harbor, ME, USA) were used at 12–14 weeks of age (17.5–21.5 g) at the time of irradiation. Mice were housed four to five per cage in a facility accredited by the Association for Assessment and Accreditation of Laboratory Animal Care International. Animal rooms were maintained at 21 ± 2  C, 50% ± 10% humidity, and 12-h light/dark cycle. Commercial rodent ration (Harlan Teklad Rodent Diet 8604) and acidified water (pH = 2.5–3.0) to control opportunistic infections^50^ were freely available.

Mice received total body irradiation (TBI), at a 0.615 Gy/min dose rate in a bilateral gamma radiation field at AFRRI’s ^60^Co facility as previously described^39^. Sham irradiated mice were placed in jigs for the same time periods as mice that were irradiated, but did not receive radiation. For survival studies, statistical studies have demonstrated that n = 16–20 per group is sufficient for statistical analysis^51^. Captopril, 0.55 g/L or 0.065 g/L (USP grade, Sigma-Aldrich, St. Louis, MO, USA), was dissolved in acidified water. A previous study established captopril stability in acidified water^52^. Captopril consumption was calculated based on volume of water consumed daily and body weights over the time course of the experiment; water intake is reduced in days 0–4 post-irradiation and is maximal at 22–30 days post-irradiation^16^. Captopril at 0.55 g/L in the water was calculated to result in maximal delivery of 58–110/kg/day, and captopril at 0.065 g/L was calculated to result in maximal delivery of 6.8–13 mg/kg/day. Vehicle treated animals received acidified water with no drug added. Mice were anesthetized with pentobarbital and blood was obtained by cardiocentisis, as previously described^13^. Complete blood counts (CBC) with differentials were obtained using a Baker Advia 2120 Hematology Analyzer (Siemens, Tarrytown, NY, USA). Separate mice were used at each time point (n = 5–6).

### Histology and pathology scoring

Sternabrae and brains were removed after euthanasia. Sternabrae included small portions of rib segments and placed in 10% formalin overnight. Samples were moved to 70% ethanol and transferred to Histoserv, Inc. (Germantown, MD, USA) for decalcification, paraffin embedding, and staining with H&E and Masson’s. Sternabrae sections were scored by a pathologist in a blinded fashion to determine cellularity^53^.

Brains were fixed in formalin for 48 h, washed in PBS and trimmed rostro-caudally into 5 blocks of tissue. Blocks approximately 2 mm thick starting from +2.22 mm Bregma to −7.5 mm were stored in 50% ethanol and sent to Histoserv, Inc. for processing and embedding in paraffin. 5 µm thick paraffin sections were stained for H&E (Histoserv, Inc.) and scored by a pathologist in a blinded fashion using criteria for level 1–4 hemorrhage severity^54^. One section per block was imaged on a Nikon eclipse Ti microscope, using a Nikon Ds-Ri2 camera and NIS-elements AR 4.4 software. Hemorrhages in the brain were counted (n = 4–5 per group) and the areas of the hemorrhages were quantified using the NIS-elements software.

### Gene expression analysis

Bone marrow was harvested by aspiration of femurs after euthanasia and placed in RNA*later* (Qiagen, Valencia, CA, USA). Samples were homogenized further by filtering through QIAshredder mini columns (Qiagen) for 3 min at 16,000 × *g*. RNA was isolated from 350 μl filtrate using the RNeasy kit according to the manufacturer’s protocol (Qiagen). Genomic DNA was removed using the RNase-Free DNAse Set (Qiagen). RNA was quantified spectroscopically (ND-1000 Spectrophotometer, NanoDrop Technologies, Wilmington, DE, USA). For cDNA, 1.0 μg of RNA was reverse transcribed using iScript cDNA Syntehsis Kit according to the manufacturer’s protocol (BioRad, Hercules, CA, USA). Quantitative real-time polymerase chain reactions (qRT-PCR) used cDNA (diluted 1:10 in 20 μl). Primer pairs and product lengths are provided in Table 1. qRT-PCR was performed in duplicate. For a 10 µl qPCR reaction, 2.5 µl of cDNA was used as a template, after diluting cDNA synthesis reaction to 100 µl, with 1 µM of each primer and 5 μl of iTaq universal SybrGreen PCR supermix (Bio-Rad). PCR reaction conditions were: 95  C for 3 min followed by 40 cycles of 95 °C for 10 sec and 55 °C for 30 sec. Absence of non-specific amplification was confirmed by melting curve analysis. The comparative threshold cycle (Ct) method was used to assess relative changes in mRNA levels using endogenous hypoxanthine-guanine phosphoribosyltransferase (HPRT) as an internal reference. Data were collected from 4 to 6 replicates unless indicated.

**Table 1:** Primers for qPCR.

Gene	Forward Primer	Reverse Primer
p15 CDkn2b	5′-AGATCCCAACGCCCTGAAC-3′	5′-CAGTTGGGTTCTGCTCCGT-3′
p16 CDkn2a	5′-GGGTTTTCTTGGTGAAGTTCG-3′	5′-TTGCCCATCATC ATCACCT-3′
p21 CDkn1a	5′-TCCACAGCGATATCCAGACA-3′	5′-GGACATCACCAGGATTGGAC-3′
HPRT	5′-CACAGGACTAGAACACCTGC-3′	5′-GCTGGTGAAAAGGACCTCT-3′

### Serum cytokine levels

Mouse serum samples were obtained by cardiocentesis following euthanasia. Samples were aliquoted and frozen at −80 °C until analysis. Mouse serum was assayed in technical duplicates with minimum of three biological repeats using either standard ELISAs (R&D Systems, Minneapolis, MN, USA) or using the electrochemiluminescent MesoScale Discovery (MSD) UPlex (MesoScale Discovery, Gaithersburg, MD, USA). ELISAs were performed according to the manufacturer’s instructions with technical duplicates and standard controls for murine FMS-like tyrosine kinase 3 (FLT-3) ligand, granulocyte colony-stimulating factor (G-CSF), stem cell factor (SCF), and serum amyloid A1 (SAA1). MSD Uplex plates were used to quantitatively measure 9 cytokines, including murine erythropoietin (EPO), interleukin (IL)-1β, IL-2, IL-6, IL-10, keratinocyte chemoattractant/chemokine (C-X-C motif) ligand 1 (KC/CXCL1), monocyte chemoattractant protein 1 (MCP1), and macrophage inflammatory protein (MIP) 1a and 1b. All assays were performed according to the manufacturer’s instructions with standard controls. The data were acquired on the MSD QuickPlex SQ120 plate reader and analyzed using the Discovery Workbench 3.0 software (MSD). The standard curves for each cytokine were generated using the premixed lyophilized standards provided in the kits. Serial 4-fold dilutions of the standards were run to generate a 7-standard concentration set, and the diluent alone was used as a blank. The cytokine concentrations were determined from the standard curve using a 4-parameter logistic fit curve to transform the mean light intensities into concentrations. The lower limit and upper limit of quantification was determined for each cytokine and all but one sample values fell within the detection ranges of the assays. Those within the detection ranges showed <10% Calc. Conc. CVs.

### Statistical analysis

Kaplan–Meier plots were analyzed using Fisher Exact Tests to assess the differences in survival between the groups after irradiation (GraphPad Prism v7.1, LaJolla, CA, USA). *P* values lower than 0.05 were considered to be statistically significant. Plasma cytokine levels, hematology results, and gene expression changes were analyzed using two way ANOVA followed by Holm-Sidak or Tukey postanalysis (GraphPad Prism v7.1). A value of *p* ≤ 0.05 (two-tailed) was considered statistically significant.

## Supplementary information

Supplementary information
