# Supplementary material for: Delayed Captopril Administration Mitigates Hematopoietic Injury in a Murine Model of Total Body Irradiation
Source: Sci Rep. 2019 Feb 18;9:2198. doi: 10.1038/s41598-019-38651-2 (PMC6379397; doi:10.1038/s41598-019-38651-2)
Supplement: Supplementary file 1 — Supplementary information [file 41598_2019_38651_MOESM1_ESM.pdf]

Delayed Captopril Administration Mitigates Hematopoietic Injury in a Murine Model of Total Body Irradiation

Elizabeth A. McCart[1*], Young H. Lee[2*], Jyoti Jha[3], Ognoon Mungunsukh[4], W. Bradley Rittase[1], Thomas A. Summers, Jr.[5], Jeannie Muir[5], Regina M. Day[1]

[1]Department of Pharmacology and Molecular Therapeutics, Uniformed Services University of the Health Sciences, Bethesda, MD 20814

[2]current address: Altimmune, Inc., Gaithersburg, MD 20878

[3]current address: Rise Therapeutics, Rockville, MD 20850

[4]current address: Department of Anesthesia, Uniformed Services University of the Health Sciences, Bethesda, MD 20814

[5]Department of Pathology, Uniformed Services University of the Health Sciences, Bethesda, MD, USA

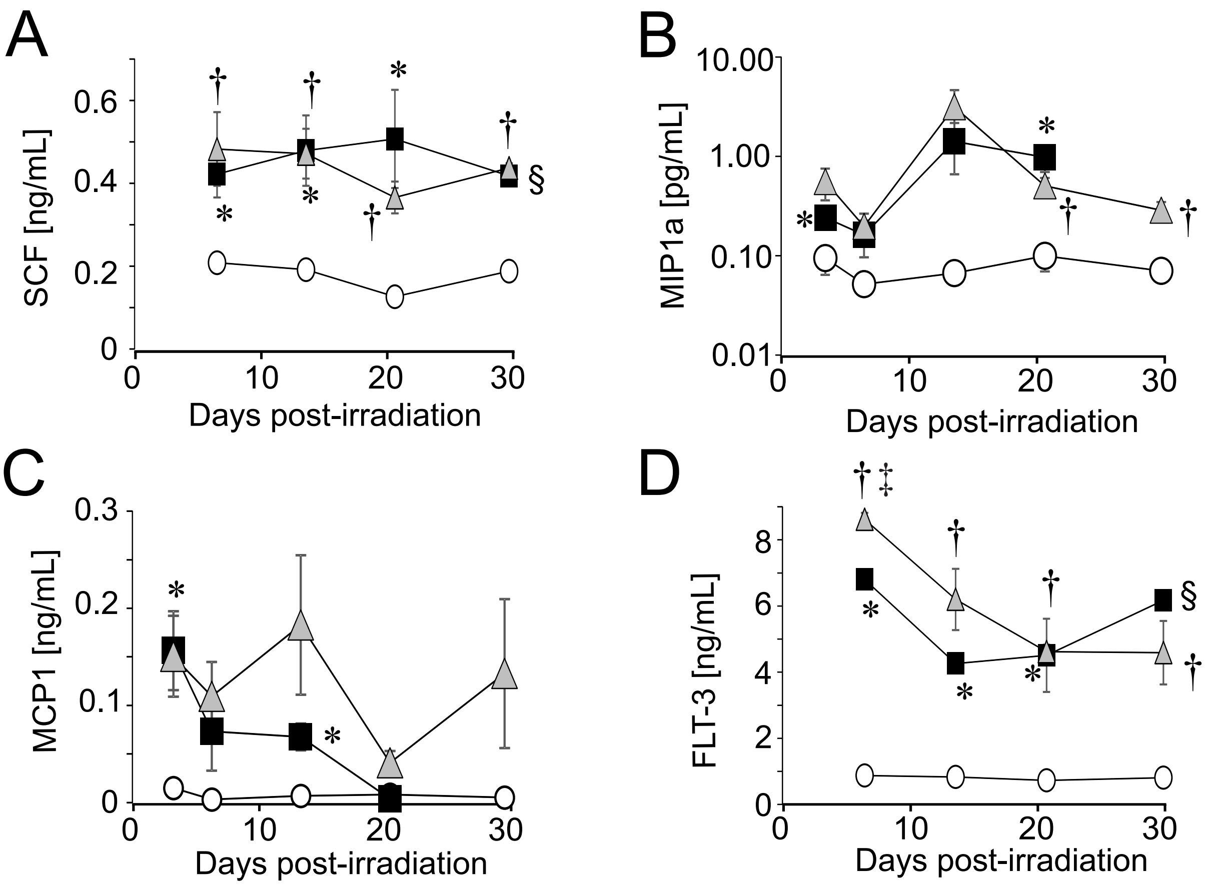

Figure S1. Effect of delayed captopril administration on cytokine levels in peripheral blood following total body irradiation. C57BL/6 mice, 12-14 weeks of age, were exposed to 7.9 Gy total body ^{60}Co irradiation (0.6 Gy/min) or sham irradiated (sham). Mice received vehicle (7.9 Gy + vehicle) or received captopril (13 mg/kg/day, 7.9 Gy + Cap), administered in the drinking water 48 h – 14 days post-irradiation. Serum was obtained at the indicated times and growth factors and cytokines were determined by MSD or ELISA. A. SCF; B. MIP1a; C. MCP1; D. FLT-3. Data show means ± SEM, n = 3-5 per group, except for the 30 day time point for radiation + vehicle, which had in some cases had no animals left or only one animal (indicated by §). * indicates $p < 0.05$ between radiation + vehicle and sham; † indicates $p < 0.05$ between radiation + captopril and sham; ‡ indicates $p <0.05$ between radiation + vehicle and radiation + captopril.

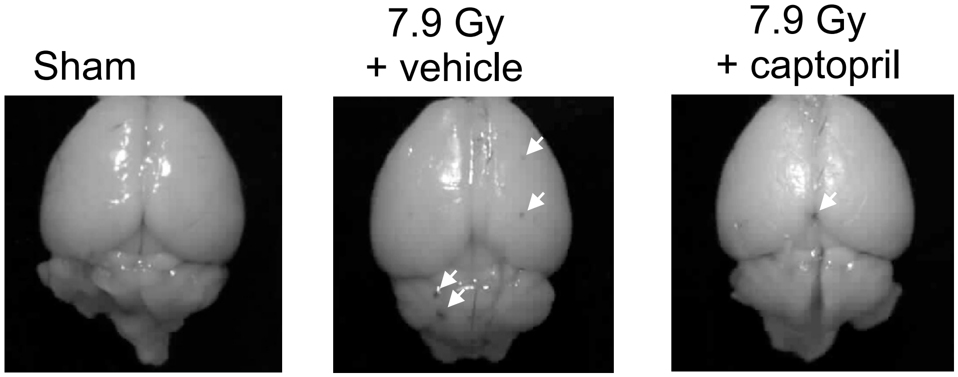

Figure S2. Captopril reduces brain hemorrhage following 7.9 Gy total body irradiation at 21 days post-irradiation. C57BL/6 mice, 12-14 weeks of age, were exposed to 7.9 Gy total body ^{60}Co irradiation (0.6 Gy/min) or sham irradiated (sham). Mice received vehicle (7.9 Gy + vehicle) or received captopril (13 mg/kg/day, 7.9 Gy + Cap), administered in the drinking water 48 h – 14 days post-irradiation. At 21 days post-irradiation, brains were obtained after euthanasia and fixed for histology. Images show dorsal views of intact mouse brains for the three treatment groups. Representative brains are shown; white arrows indicate hemorrhages.

Table S1. Bone marrow cellularity scores.

	Days post-irradiation			
	7	14	21	30
Sham	99<br>99<br>99<br>99	99<br>99<br>99<br>99	99<br>99<br>99<br>99	99<br>99<br>99<br>99
Radiation + vehicle	0.5<br>0.5<br>0.5<br>0.5<br>0.5	0.5<br>0.5<br>0.5<br>1.0<br>0.5	0.5<br>20<br>0.5	30
Radiation + captopril	11<br>5.0<br>1.0<br>1.0<br>1.0	1.0<br>1.0<br>5.0<br>10.0<br>1.0	1.0<br>70<br>5.0<br>5.0	90<br>95<br>99<br>85

C57BL/6 mice, 12-14 weeks of age, were exposed to 7.9 Gy total body ^{60}Co irradiation (0.6 Gy/min) or sham irradiated (sham). Mice received vehicle (Radiation + vehicle) or received captopril (13 mg/kg/day, Radiation + Captopril), administered in the drinking water either 48 h through 14 days post-irradiation. Bone marrow cellularity was scored by a hematological pathologist blinded to the treatment groups, n = 3-5 per group, except for the 30 day time point for radiation + vehicle, which had only one animal.
